# Staging of rat liver fibrosis using monoexponential, stretched exponential and diffusion kurtosis models with diffusion weighted imaging- magnetic resonance

**DOI:** 10.18632/oncotarget.23413

**Published:** 2017-12-18

**Authors:** Genwen Hu, Wen Liang, Mingxiang Wu, Queenie Chan, Yufa Li, Jianmin Xu, Liangping Luo, Xianyue Quan

**Affiliations:** ^1^ Department of Radiology, The Second Clinical Medical College of Jinan University, Shenzhen People's Hospital, Shenzhen 518020, China; ^2^ Department of Radiology, Zhujiang Hospital, Southern Medical University, Guangzhou 510282, China; ^3^ MR Clinical Science, Philips Healthcare, Hong Kong 20023, China; ^4^ Department of Pathology, Guangdong General Hospital, Guangdong Academy of Medical Sciences, Guangzhou 510080, China; ^5^ Department of Radiology, The First Affiliated Hospital of Jinan University, Guangzhou 510280, China

**Keywords:** liver fibrosis, diffusion weighted imaging, stretched exponential model, diffusion kurtosis imaging

## Abstract

Early diagnosis of liver fibrosis is important. The objective of this study was to explore the characteristics and to assess the accuracy of monoexponential, stretched exponential models (SEM), and diffusion kurtosis imaging (DKI) with diffusion-weighted imaging (DWI)-magnetic resonance imaging (MRI) in various stages of liver fibrosis in two standard rat models induced by carbon tetrachloride (CCl_4_) and biliary duct ligation (BDL). Parameters (ADC, D_app_, K_app_, DDC, α) were measured with a 3.0T MRI. Liver fibrosis stages (F0–F4) were defined by METAVIR scoring. Parameters (ADC, D_app_, DDC) were found to be negatively associated (r: -0.675~-0.789; *P*<0.05) with advancement of fibrosis stage. The analysis of receiver operating characteristic (ROC) curves illustrated that the areas under the curves (AUC) for ADC, D_app_, and DDC were 0.687~0.957, 0.805~0.938 and 0.876~1.000, respectively. The study showed that (ADC, D_app_, K_app_, DDC, α) from various diffusion models reflected pathological and physiological tissue changes. We conclude that SEM and DKI may provide more accurate information about diffusion, and non-Gaussian diffusion analysis may be a complementary tool for the assessment of liver fibrosis.

## INTRODUCTION

Liver fibrosis is a reparative response of liver tissues to the chronic liver injuries of various causes. When the rate of synthesis of extracellular matrix (ECM) exceeds that of degradation, distortion of liver lobules and blood circulation disorders due to cirrhosis can occur. Early fibrosis is reversible, [1, 2] and its progression into cirrhosis and cancer can be stopped or delayed by early treatment. Thus, early diagnosis of liver fibrosis is clinically important.

There are three types of diagnostic methods for liver fibrosis currently being used clinically: percutaneous biopsy, serological tests, and imaging. The standard method for diagnosis of liver fibrosis is biopsy; however, this is invasive and not suitable for repeated examinations or dynamic observations [3]. The application of serological testing is largely limited by low sensitivity and specificity [4, 5].

Conventional imaging is not sufficiently accurate to diagnose liver fibrosis [6]. Ultrasound elastography is currently considered the most appropriate means of diagnosing liver fibrosis; however, MRI can also provide a valuable reference for diagnosis. Magnetic resonance diffusion-weighted imaging (MR-DWI) reflects the random Brownian motion of water molecules diffusing in biological media. Measurement of degrees of partial restriction as well as direction during water molecule diffusion can indirectly reflect changes in tissue microstructure. In clinical studies, cirrhotic livers possessed significantly lower apparent diffusion coefficient (ADC) values in a monoexponential model compared with non-fibrotic livers [7–9]. However, in addition to the intra-tissue diffusion of water molecules, ADC is also influenced by the microcirculation of the blood in capillaries [10–12].

Intravoxel incoherent motion (IVIM) from the biexponential model is an advanced form of DWI that can measure both diffusion and perfusion. IVIM more accurately reflects the pathological and physiological changes of tissues [13, 14]. However, studies have shown that the IVIM technique cannot offer reliable measurements for staging liver fibrosis [15].

The monoexponential model is based on the characterization of the Gaussian diffusion of water molecules. [16] However, water diffusion behavior *in vivo* is more complex than Gaussian diffusion due to the presence of various diffusion barriers such as membranes in the sophisticated cellular structures of tissues. Therefore, advanced non-Gaussian diffusion models, such as the stretched exponential model (SEM) and diffusion kurtosis imaging (DKI), may provide more accurate information about real water molecular motion *in vivo* [17].

The current study compared monoexponential and stretched exponential models, as well as diffusion kurtosis MR staging with histological staging in a rat model of liver fibrosis. We computed the parameters (ADC, D_app_, K_app_, DDC, α) of liver fibrosis model at different pathologic stages: ADC from monoexponential; D_app_, K_app_ from DKI; and DDC, α from SEM. We also evaluated and compared the characteristics of these parameters for the detection and evaluation of liver fibrosis.

## RESULTS

### Fibrosis model establishment and staging

**Figure 1:**
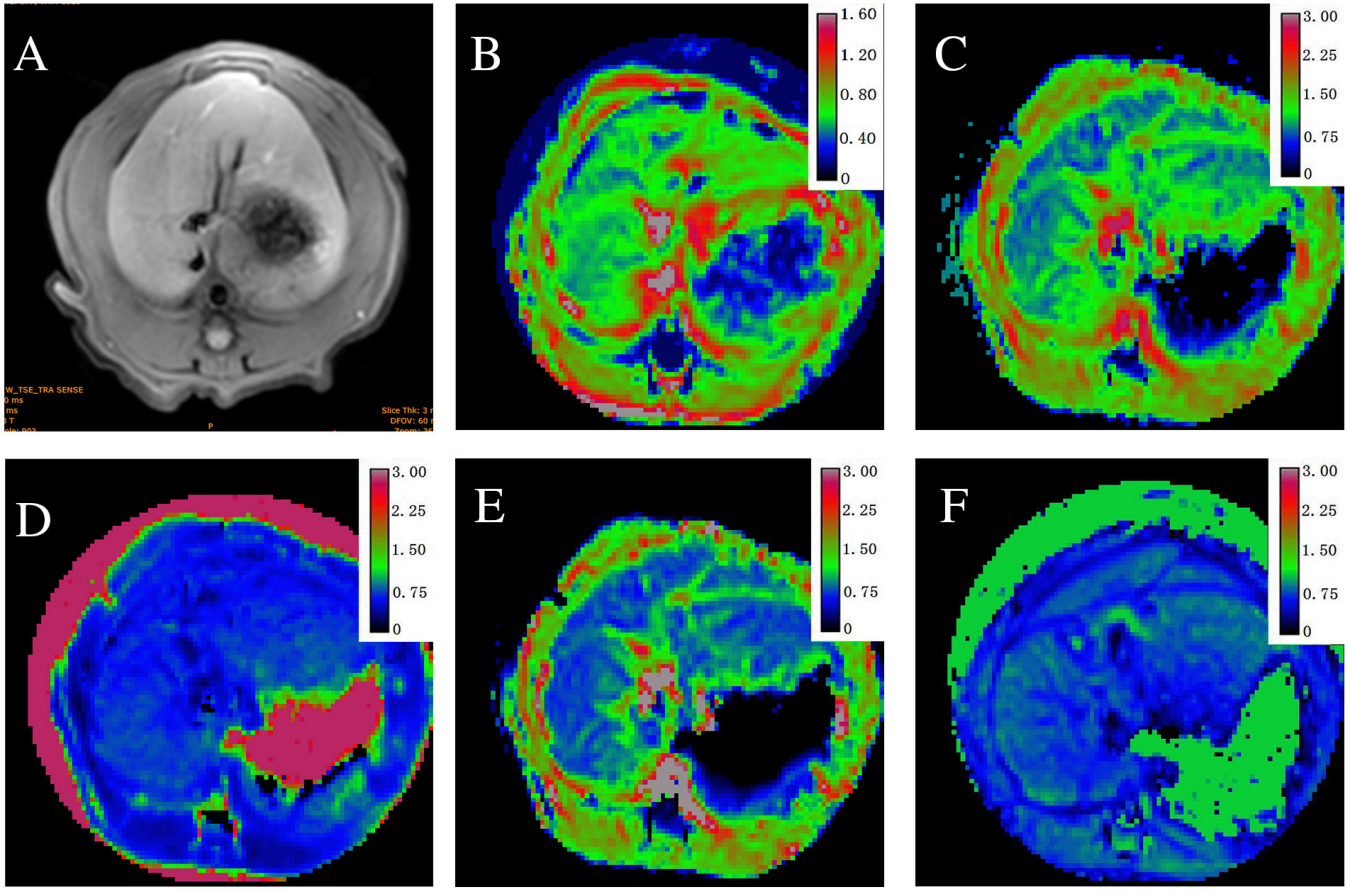
Axial MR images in sequence from a stage F2 fibrosis induced by carbon tetrachloride (CCl4) **(A)** T1-weighted image; **(B)** mean values (0.92×10^−3^ mm^2^/s) shown by a map of apparent diffusion coefficients (ADC); **(C)** mean values (1.10×10^−3^ mm^2^/s) shown by the D_app_ map; **(D)** mean values (1.02) shown by the K_app_ map; **(E)** mean values (0.76×10^−3^ mm^2^/s) shown by the DDC map; **(F)** mean values α (0.68) shown by a map.

**Figure 2:**
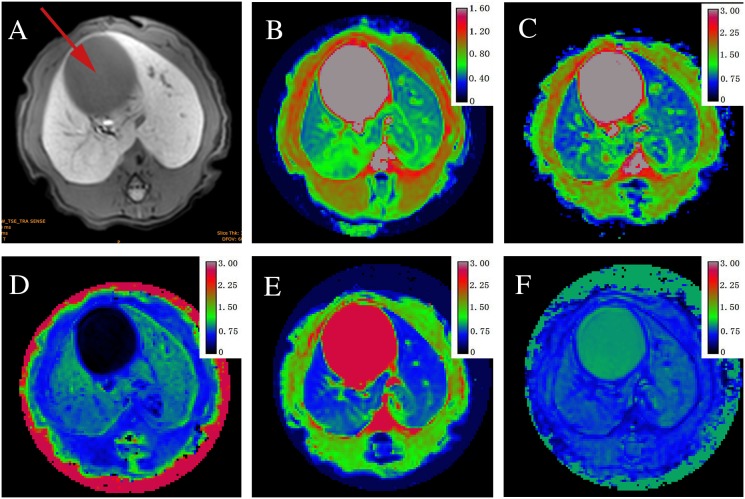
Axial MR images in sequence from a stage F2 fibrosis induced by biliary duct ligation (BDL) **(A)** T1-weighted image, red arrow = dilated biliary duct; **(B)** mean values (0.88×10^−3^ mm^2^/s) shown by a map of apparent diffusion coefficients (ADC); **(C)** mean values (0.99×10^−3^ mm^2^/s) shown by the D_app_ map; **(D)** mean values (1.07) shown by the K_app_ map; **(E)** mean values (0.68×10^−3^ mm^2^/s) shown by DDC map; **(F)** mean values α (0.64) shown by map.

**Figure 3:**
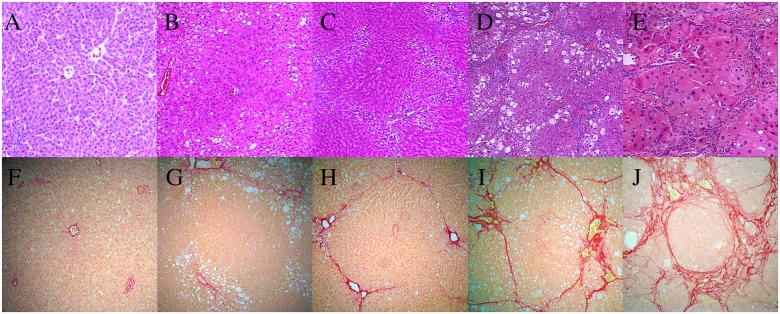
Typical hematoxylin and eosin (H&E) staining and picrosirius red staining for representative samples in the carbon tetrachloride (CCl4) group **(A–E)** Liver fibrosis stages F0–F4 (H&E; 10×20), **(F–J)** Liver fibrosis stages F0–F4 (picrosirius red; 10×20).

MR images shown in Figure 1 and Figure 2 No fibrosis occurred in the control group (F0, N=16). In the carbon tetrachloride (CCl_4_) group, as shown in Figure 3, pathological evaluations revealed 8 rats as stage F1, 7 rats as stage F2, 8 rats as stage F3, and 9 rats as stage F4 liver fibrosis.

**Figure 4:**
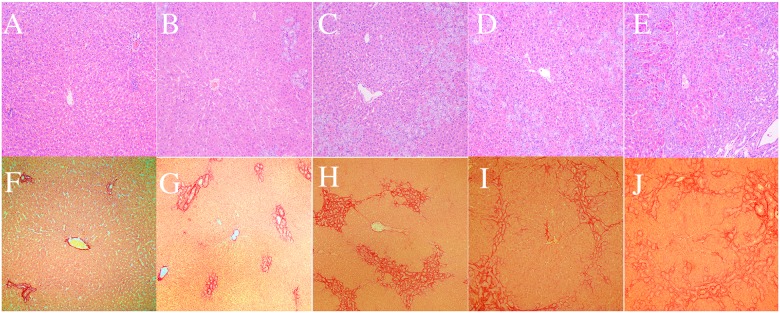
Typical hematoxylin and eosin (H&E) staining and picrosirius red staining for representative samples in the biliary duct ligation (BDL) group **(A–E)** Liver fibrosis stages F0–F4 (H&E; 10×20), **(F–J)** Liver fibrosis stages F0–F4 (picrosirius red; 10×20).

In the biliary duct ligation (BDL) group, as shown in Figure 4, pathological evaluations revealed that 8 rats had stage F1, 9 rats had stage F2, 9 rats had stage F3, and 6 rats had stage F4 fibrosis.

### Change of parameters

The diffusion parameters (ADC, D_app_, DDC) decreased with increasing fibrosis levels. With the application of the LSD *post hoc* test, all diffusion parameters in fibrotic livers (F1–F4) were significantly lower than those in normal rat livers (F0) (*P*<0.05). The diffusional kurtosis (K_app_) and water molecular diffusion heterogeneity index (α) increased with increasing fibrosis levels. However, the data overlapped (Table 1).

**Table 1:** Summary of mean values and standard deviations for the parameters (ADC, D_app_, K_app_, DDC, α) in various liver fibrosis stages

Fibrosis stage	N (80)	ADC (×10^−3^ mm^2^/s)	D_app_ (×10^−3^ mm^2^/s)	K_app_	DDC (×10^−3^ mm^2^/s)	α
0	16	1.283±0.138	1.581±0.192	0.930±0.083	1.353±0.268	0.569±0.073
1	16	1.056±0.151	1.343±0.183	0.956±0.080	1.101±0.173	0.600±0.071
2	16	0.915±0.131	1.090±0.171	1.025±0.093	0.761±0.147	0.687±0.075
3	17	0.863±0.109	1.072±0.160	1.065±0.085	0.697±0.129	0.674±0.067
4	15	0.884±0.088	0.992±0.095	1.070±0.083	0.679±0.146	0.642±0.066
*P* value		<0.001	<0.001	<0.001	<0.001	<0.001

### Parameter correlations with histologically stage of liver fibrosis and the collagen content

The Spearman rank correlation test showed a negative correlation (r: -0.675~-0.789; *P*<0.05) between fibrosis stage and both of the diffusion parameters (ADC, D_app_, DDC). Conversely, K_app_ and the α parameters had positive correlations (r: 0.365~0.549, *P*<0.05), although the “r” is low. (Table 2)

**Table 2:** Spearman correlations and 95% confidence intervals (CI) between fibrosis stages and imaging parameters

Model	Parameters	Fibrosis stage	*P* value
Monoexponential	ADC	-0.675 (-0.781, -0.522)	<0.001
Diffusion	D_app_	-0.743 (-0.825, -0.621)	<0.001
Kurtosis	K_app_	0.549 (0.386, 0.690)	<0.001
Stretched	DDC	-0.789 (-0.859, -0.671)	<0.001
Exponential	α	0.365 (0.158, 0.548)	<0.001

The Pearson rank correlation test showed a negative correlation (r: -0.559~-0.617; P<0.05) between collagen content and both of the diffusion parameters (ADC, D_app_, DDC). Conversely, K_app_ and the α parameters had weak positive correlations (r: 0.268~0.459, P<0.05). (Table 3)

**Table 3:** Pearson correlations and 95% confidence intervals (CI) between the percentage of collagen area and imaging parameters

Model	Parameters	Percentage of collagen area	*P* value
Monoexponential	ADC	-0.559 (-0.652, -0.454)	<0.001
Diffusion	D_app_	-0.617 (-0.705, -0.521)	<0.001
Kurtosis	K_app_	0.459 (0.282, 0.619)	<0.001
Stretched	DDC	-0.601 (-0.682, -0.521)	<0.001
Exponential	α	0.268 (0.093, 0.429)	<0.001

### ROC curve analyses

In the carbon tetrachloride (CCl_4_) group, an analysis of ROC curves for fibrosis stage evaluation showed that the AUC of ADC for any (≥stage1), significant (≥stage 2) or advanced fibrosis (≥stage 3) and cirrhosis was 0.898, 0.924, 0.795 and 0.685, respectively. The AUC of D_app_ was 0.957, 0.982, 0.836 and 0.824, respectively. The AUC of DDC was 0.953, 0.984, 0.880 and 0.781, respectively.

In the biliary duct ligation (BDL) group, an analysis of ROC curves for fibrosis stage evaluation showed that the AUC of ADC for any (≥stage 1), significant (≥stage 2) or advanced fibrosis (≥stage 3) and cirrhosis was 0.988, 0.878, 0.860 and 0.750, respectively. The AUC of D_app_ was 0.922, 0.891, 0.844 and 0.770, respectively. The AUC of DDC was 0.922, 0.969, 0.877 and 0.804, respectively.

**Figure 5:**
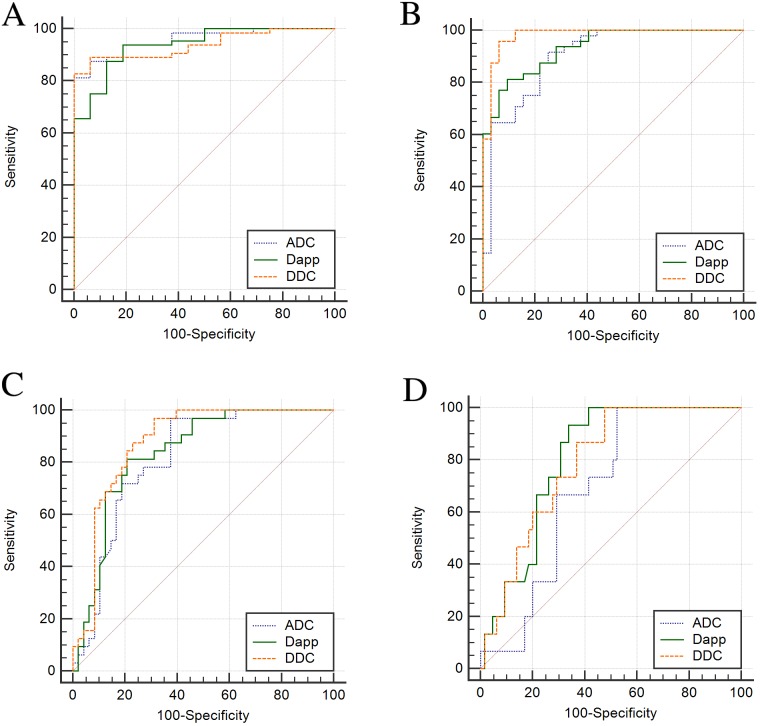
Graph showing results of receiver operator characteristic (ROC) analysis based on diffusion parameters of METAVIR stages **(A)** (ADC, D_app_, DDC) in F0 vs. F1–4. **(B)** (ADC, D_app_, DDC) in F0–1 vs. F2–4, **(C)** (ADC, D_app_, DDC) in F0–2 vs. F3–4, **(D)** (ADC, D_app_, DDC) in F0–3 vs. F4.

Taken together, Table 4 and Figure 5 demonstrates the ROC curves for differentiating fibrosis stages with the parameters ADC, D_app_, and DDC. As shown in Table 3, the ROC curves summarize the optimal cut-off values, producing the sensitivity and specificity for distinguishing between each fibrosis stage. Analysis of ROC curves for fibrosis stage evaluation showed that the AUC for D_app_ and DDC were 0.805~0.938 and 0.876~1.000, respectively, which were higher than ADC (0.687~0.957), except for the control group versus the fibrosis group (F0 versus F1-2-3-4).

**Table 4:** Diagnostic accuracy of values across METAVIR stages

	AUC	95% CI	Cut-off value	Sensitivity	Specificity
F0 versus F1-2-3-4					
ADC	0.957	0.887 - 0.990	≤ 1.0.5	81.25	100
D_app_	0.938	0.860 - 0.979	≤1.352	87.50	87.50
DDC	0.938	0.861 - 0.980	≤1.022	82.81	100.00
F0-1 versus F2-3-4					
ADC	0.902	0.815 - 0.957	≤ 1.035	91.67	75.00
D_app_	0.933	0.854 - 0.977	≤1.155	81.25	90.62
DDC	0.980	0.921 - 0.998	≤0.929	95.83	93.75
F0-1-2 versus F3-4					
ADC	0.813	0.710 - 0.891	≤ 1.010	96.87	62.50
D_app_	0.835	0.735 - 0.909	≤1.104	81.25	79.17
DDC	0.876	0.783 - 0.939	≤0.916	96.87	68.75
F0-1-2-3 versus F4					
ADC	0.687	0.574 - 0.786	≤ 1.01	100.00	47.69
D_app_	0.805	0.701 - 0.885	≤1.104	93.33	66.15
DDC	1.000	0.955 – 1.000	≤0.987	100	100

## DISCUSSION

In this work, we employed the stretched exponential model and diffusion kurtosis imaging to characterize the non-Gaussian behavior of the diffusion-related signal decay in two animal models of liver fibrosis. Because the two sets of data tended to be consistent, we addressed them together in the discussion.

MR-DWI is a functional MRI technique that reflects the random Brownian motion of water molecular diffusion in biological media analyzed by conventional ADC from the monoexponential model. SEM and DKI are emerging methods from a non-Gaussian distribution model with DWI. DKI includes parameters D_app_ (the ADC after non-Gaussian correction) and K_app_ (the mean kurtosis, a dimensionless parameter reflecting the deviation of diffusion distribution from the Gaussian form). SEM has the parameters DDC (the distributive diffusion coefficient, which is similar to the standard diffusion coefficient) and α (the water molecular diffusion heterogeneity index). α close to 1 indicates low heterogeneity of intra-tissue diffusion, while α close to 0 indicates high heterogeneity.

The diffusion parameters (ADC, D_app_, DDC) decreased with increasing fibrosis levels in the current study. Previous studies showed that the ADC declined after the development of liver fibrosis [7–9]. This has been generally explained by the fact that during liver fibrosis, liver damage results in necrosis, apoptosis, and inflammation in liver cells. This could result in the secretion of various cytokines and lipid superoxides that jointly act on hepatic stellate cells to synthesize ECMs. ECM deposition as well as leakage of fluids from liver cells and infiltration of inflammatory cells during liver fibrosis can restrict water molecule diffusion and lead to the reduction of the diffusion parameters (ADC, D_app_, DDC) [18, 19].

Correlation was found between fibrosis stages and diffusion parameters. Collagen content and diffusion parameters both showed that D_app_ and DDC from the non-Gaussian model were superior to the r of ADC. This illustrates that the non-Gaussian model was more consistent with the real status of diffusion in liver fibrosis tissue likely due to the presence of various diffusion barriers such as ECMs, inflammation, hepatocyte ballooning, and steatosis.

Although the exact significance of α and K_app_ *in vivo* remain not fully understood, some reports indicated that α may reflect microstructural heterogeneity [17] and K_app_ may indicate microstructural complexity [20].

The current study showed that K_app_ increased with increasing fibrosis levels. This may be partly explained by the complicated hepatic microstructure in the presence of fibrosis, although there was overlap in the varying degrees of fibrosis. Our results are similar to those in the latest reports on the subject [21].

Regarding α, a previous study reported that α is a useful marker in grading of gliomas [22]. Few reported studies have investigated the use of α in liver fibrosis, and α is regarded to be unrelated to pathological grading [18]. However, that report was limited to *in vitro* studies. The current study showed that α increased in fibrosis compared to normal liver tissue.

The use of ADC to evaluate the stage of liver fibrosis is currently controversial. Several studies published on this topic have not shown unequivocal results [23]. In our study, ROC analysis of the parameters used in evaluating the stages of liver fibrosis showed that ADC was valuable in determining the stage of liver fibrosis. The findings in this study were similar to those reported by Fujimoto [24] and superior to those of Bonekamp [25]. However, the current study indicated that the non-Gaussian parameters D_app_ and DDC had better predictive values than ADC with regard to determining the stage of liver fibrosis, with the exception of the control groups versus fibrosis group (F0 versus F1-2-3-4). This indicates that for the AUC of F0 versus F1-2-3-4, ADC was better, but in others, D_app_ and DDC was better than ADC, for example, in distinguishing between mild and severe fibrosis (F0-1-2 versus F3-4) and between non-cirrhosis and cirrhosis (F0-1-2-3 versus F4).

ADC may correlate with water motion, which is also influenced by the concentration of macromolecules and microcirculation of blood. While D_app_ and DDC not only potentially better reflect water diffusivity in tissues at ultrahigh b values, they also contain specific information on the non-Gaussian diffusion behavior in addition to the intra-tissue diffusion of water molecules [21].

This study uses rat models to study the imaging changes in human liver fibrosis. No current animal model can completely and accurately reflect human liver fibrosis. Notably, some animal models may be more suited for testing well defined hypotheses. It is also possible that the diagnostic performance of techniques differs between animals and humans. A non-Gaussian diffusion model employed a high b-value diffusion, which extended scanning time and included effects of respiratory movement that needed to be considered. Thus, the reproducibility of the DWI biomarkers should be studied further.

One of the main limitations of the conclusion that SEM and DKI are superior to ADC is that the conclusion was based only on the finding that the AUC of the ROC were higher. More studies with stronger statistics will be needed to confirm these results. Other limitations are the lack of analysis of stellate cell activation and the use of a semiquantitative analysis of collagen content.

In summary, this study analyzed (via rat models) various diffusion models in liver fibrosis. Each parameter reflects the pathological and physiological changes in the corresponding tissues. The diffusion parameters (ADC, D_app_, DDC) correlated strongly with the degrees of liver fibrosis. The parameters K_app_ and α may provide complementary information. The non-Gaussian parameters D_app_ and DDC were superior to ADC as diagnostic markers for liver fibrosis staging. SEM and DKI may provide more accurate information about diffusion in liver fibrosis and may be an effective complementary tool to the standard DWI monoexponential model. However, their clinical utility in the clinical evaluation of liver fibrosis remains to be evaluated.

## MATERIALS AND METHODS

### Animals and treatment

In this experiment, 80 adult male Sprague-Dawley (SD) rats (250±20 g) were obtained from Southern Medical University Laboratory Animal Co., Ltd. (Guangzhou, China). Free access to standard feed and water were provided. All of the rats were individually housed at a stable temperature (18–20°C) and humidity (60–70%) with a 12 h light/dark cycle. All of the experimental procedures were approved by the Institutional Animal Ethics Committee of our University and performed according to the Laboratory Animal Care and Usage Manual of our institute.

The rats were randomized into a liver fibrosis group (n=64) with two standard rat models induced by carbon tetrachloride (CCl_4_) (n=32) and biliary duct ligation (BDL) (n=32), as well as a control group (n=16).

Liver fibrosis was induced by carbon tetrachloride (CCl_4_) as described previously [19]. After an acclimatization period of 1 week under standard conditions, the rats in the CCl_4_ group received 50% CCl_4_ (CCl_4_: olive oil, 1:1) subcutaneously at a dose of 0.3 ml/100 g of body weight twice per week for up to 9 weeks. To obtain data on various stages of fibrosis, 4 rats were randomly selected from the liver fibrosis group for MRI at 8 time points (2, 3, 4, 5, 6, 7, 8, 9 weeks) after the first CCl_4_ injection.

In the biliary duct ligation (BDL) group [26], the rats were anesthetized with 3% pentobarbital intraperitoneal injection at a dose of 0.2 ml/100 g of body weight. An upper abdominal incision was achieved using a sterile technique, and the common bile duct was isolated and double-ligated close to the liver, immediately below the bifurcation. To obtain data on various stages of fibrosis, 8 rats were randomly selected from the liver fibrosis group for MRI at 4 time points (1, 2, 3, 4 weeks) after the operation.

### Conventional MRI techniques

An INGENIA 3.0T (Philips Healthcare, Best, Netherlands) magnetic resonance scanner with a 30-mm rat coil (Suzhou Medcoil Healthcare Co., Ltd) with 4 independent channels and a high signal to noise ratio and high resolution was used for small animal imaging. Imaging range: F/H 80 mm, R/L 47 mm, A/P 47 mm. After the rats were anesthetized with 3% pentobarbital intraperitoneal injection at a dose of 0.2 ml/100 g of body weight, MRI scans were performed in a prone and head-first position. To limit the respiratory motion of the rats, a fixing belt was used. The following conventional sequences were performed: (A) axial T2-weighted fast field echo (FFE) [repetition time/echo time (TR/TE) = 206/9.2 ms; FOV = 60×60 mm, matrix = 100×100; slice thickness = 3 mm], (B) axial T1-weighted turbo-spin-echo (TSE) [TR/TE = 400/10 ms, FOV=60×60 mm; matrix=120×93, slice thickness = 3 mm].

### Diffusion-weighted MRI

Diffusion-weighted MRI was implemented with a single-shot spin-echo echo-planar imaging (EPI) sequence. The resulting parameters were as follows: TR/TE = 2000/55 ms, EPI factor = 63, FOV=50×50 mm, slice thickness = 3 mm, number of slices = 9, matrix = 64×63, motion probing gradients in three orthogonal axes, spectral presaturation inversion recovery (SPIR) fat suppression. Monoexponential model b-values used: 0, 800 s/mm^2^. The number of signal averages for b-values 0 and 800 were 1 and 3, respectively. SEM and DKI models b-values used: 0, 700, 1400, 2100 s/mm^2^. The number of signal averages for b-values 0, 700, 1400 and 2100 were 1, 2, 3 and 4, respectively.

### Image analysis

Image analysis in this study was performed with the PRIDE DWI Tool version 1.5, a manufacturer-supplied software from Philips Healthcare (Best, Netherlands) and fitted on a pixel-by-pixel basis by the Levenberg–Marquardt algorithm:

1. Traditional monoexponential diffusion model [27] as

$$\frac{S_{b}}{S_{0}}=\exp(-b\;ADC)$$

where S_b_ and S_0_ are the diffusion weighted signal intensity for the diffusion gradient b-value of b and 0, respectively, and ADC is the apparent diffusion coefficient.

2. Diffusion kurtosis imaging (DKI) model [28] as

$$\ln(S)=\ln(S_{0})-b\;D_{app}+\;\frac{1}{6}\;[{\left(b\;D_{app}\right)}^{2}K_{app}]$$

where D_app_ is the diffusion coefficient and K_app_ is the diffusional kurtosis.

3. Stretched exponential model (SEM) [17] as:

$$\ln(S)=\ln(S_{0})-(b\;DDC){}^{\alpha }$$

where DDC indicates the distributed diffusion coefficient and α is the water molecular diffusion heterogeneity index (0-1).

Each pixel within the volume of interest of these parameters (ADC, D_app_, K_app_, DDC, α) was respectively calculated, and the parameters were expressed as mean values of all pixels. Five regions of interest (ROIs) of approximately 3–4 mm^2^ were manually drawn by a radiologist (LW, 21 years of liver radiology experience and also extensive experience in imaging of rats) to avoid the inclusion of liver margin, vessel or bile duct. Assessment was then subsequently performed using ImageJ software (NIH, Bethesda, MD).

### Histopathological analysis

After scanning, the rats were sacrificed by over-anesthetization with a 3% pentobarbital intraperitoneal injection. Next, the livers were excised and fixed using 10% formalin before being cut into 5-μm sections for both histological examination with hematoxylin and eosin (H&E) staining and picrosirius red staining for collagen visualization. A Leica DM2000 microscope (Leica Microsystems, Wetzlar, Germany) was used to examine the serial sections and make photographs. The stages of liver fibrosis were identified by an experienced histopathologist (LYF, 10 years experience in liver pathology) based on the METAVIR classification system, [29, 30] in which F0 = no fibrosis, F1 = portal fibrosis without septa, F2 = portal fibrosis and a few septa, F3 = numerous septa without cirrhosis, and F4 = cirrhosis. Semiquantitative analysis was performed for collagen content. Five visual fields were randomly selected (200× magnification). Image-Pro Plus v6.0 image analysis software (Media Cybernetics, Rockville, MD) was used to analyze the percentage of the positive-staining area relative to the whole area of the field [31].

### Statistical analysis

The Kolmogorov-Smirnov test was used to analyze the normality, in which the parameters resulted in an approximately normal distribution. The statistical analysis of multiple group comparisons of these parameters was carried out by a one-way analysis of variance (ANOVA). The relationships between the stage of liver fibrosis and these parameters were assessed using Spearman's correlation coefficient and collagen content and these parameters using Pearson's correlation coefficient.

SPSS v20.0 statistical software (SPSS, Chicago, IL) was used for analyzing data in the study. *P*<0.05 indicated a statistically significant difference. Receiver operating characteristic (ROC) curves were used to evaluate the usefulness of parameters for determining stages.
